# Markov model and markers of small cell lung cancer: assessing the influence of reversible serum NSE, CYFRA 21-1 and TPS levels on prognosis

**DOI:** 10.1038/sj.bjc.6690227

**Published:** 1999-03

**Authors:** J-M Boher, J-L Pujol, J Grenier, J-P Daurès

**Affiliations:** Département de Biostatistiques Epidemiologie et Recherche Clinique, Institut Universitaire de Recherche Clinique, Rue de la Cardonille, Montpellier,Cedex 5, France; Département des Maladies Respiratories, Centre Hospitalier Universitaire de Montpellier, Hôpital Arnaud de Villeneuve, Montpellier,Cedex, France; Centre Régional de Lutte contre le Cancer, Laboratoire de Radio-Analyses, Montpellier,Cedex, France

**Keywords:** Markov model, small cell lung cancer, NSE, TPS, CYFRA 21-1, tumour response, prognosis

## Abstract

High serum NSE and advanced tumour stage are well-known negative prognostic determinants of small cell lung cancer (SCLC) when observed at presentation. However, such variables are reversible disease indicators as they can change during the course of therapy. The relationship between risk of death and marker level and disease state during treatment of SCLC chemotherapy is not known. A total of 52 patients with SCLC were followed during cisplatin-based chemotherapy (the median number of tumour status and marker level assessments was 4). The time-homogeneous Markov model was used in order to analyse separately the prognostic significance of change in the state of the serum marker level (NSE, CYFRA 21-1, TPS) or the change in tumour status. In this model, transition rate intensities were analysed according to three different states: alive with low marker level (state 0), alive with high marker level (state 1) and dead (absorbing state). The model analysing NSE levels showed that the mean time to move out of state ‘high marker level’ was short (123 days). There was a 44% probability of the opposite reversible state ‘low marker level’ being reached, which demonstrated the reversible property of the state ‘high marker level’. The relative risk of death from this state ‘high marker level’ was about 2.24 times greater in comparison with that of state 0 ‘low marker level’ (Wald's test; *P* < 0.01). For patients in state ‘high marker level’ at time of sampling, the probability of death increased dramatically, a transition explaining the rapid decrease in the probability of remaining stationary at this state. However, a non-nil probability to change from state 1 ‘high marker level’ to the opposite transient level, state 0 ‘low marker level’, was observed suggesting that, however infrequently, patients in state 1 ‘high marker level’ might still return to state 0 ‘low marker level’. Almost similar conclusions can be drawn regarding the three-state model constructed using the tumour response status. For the two cytokeratin markers, the Markov model suggests the lack of a true reversible property of these variables as there was only a very weak probability of a patient returning to state ‘low marker level’ once having entered state ‘high marker level’. In conclusion, The Markov model suggests that the observation of an increase in serum NSE level or a lack of response of the disease at any time during follow-up (according to the homogeneous assumption) was strongly associated with a worse prognosis but that the reversion to a low mortality risk state remains possible. © 1999 Cancer Research Campaign


*British Journal of Cancer* (1999) **79**(9/10), 1419–1427
© 1999 Cancer Research Campaign
Article no. bjoc.1998.0227

### Markov model and markers of small cell lung cancer: assessing the influence of reversible serum NSE, CYFRA 21-1 and TPS levels on prognosis

**J-M Boher[1], J-L Pujol[1,2], J Grenier[3] and J-P Daurès[1]**

[1]Département de Biostatistiques Epidemiologie et Recherche Clinique, Institut Universitaire de Recherche Clinique, Rue de la Cardonille, 34093 Montpellier Cedex 5, France; [2]Département des Maladies Respiratories, Centre Hospitalier Universitaire de Montpellier, Hôpital Arnaud de Villeneuve, 34295 Montpellier Cedex, France; and [3]Centre Régional de Lutte contre le Cancer, Laboratoire de Radio-Analyses, 34094 Montpellier Cedex, France

**Summary** High serum NSE and advanced tumour stage are well-known negative prognostic determinants of small cell lung cancer (SCLC) when observed at presentation. However, such variables are reversible disease indicators as they can change during the course of therapy. The relationship between risk of death and marker level and disease state during treatment of SCLC chemotherapy is not known. A total of 52 patients with SCLC were followed during cisplatin-based chemotherapy (the median number of tumour status and marker level assessments was 4). The time-homogeneous Markov model was used in order to analyse separately the prognostic significance of change in the state of the serum marker level (NSE, CYFRA 21-1, TPS) or the change in tumour status. In this model, transition rate intensities were analysed according to three different states: alive with low marker level (state 0), alive with high marker level (state 1) and dead (absorbing state). The model analysing NSE levels showed that the mean time to move out of state ‘high marker level’ was short (123 days). There was a 44% probability of the opposite reversible state ‘low marker level’ being reached, which demonstrated the reversible property of the state ‘high marker level’. The relative risk of death from this state ‘high marker level’ was about 2.24 times greater in comparison with that of state 0 ‘low marker level’ (Wald’s test; $P < 0.01$). For patients in state ‘high marker level’ at time of sampling, the probability of death increased dramatically, a transition explaining the rapid decrease in the probability of remaining stationary at this state. However, a non-nil probability to change from state 1 ‘high marker level’ to the opposite transient level, state 0 ‘low marker level’, was observed suggesting that, however infrequently, patients in state 1 ‘high marker level’ might still return to state 0 ‘low marker level’. Almost similar conclusions can be drawn regarding the three-state model constructed using the tumour response status. For the two cytokeratin markers, the Markov model suggests the lack of a true reversible property of these variables as there was only a very weak probability of a patient returning to state ‘low marker level’ once having entered state ‘high marker level’. In conclusion, The Markov model suggests that the observation of an increase in serum NSE level or a lack of response of the disease at any time during follow-up (according to the homogeneous assumption) was strongly associated with a worse prognosis but that the reversion to a low mortality risk state remains possible.

**Keywords:** Markov model; small cell lung cancer; NSE; TPS; CYFRA 21-1; tumour response; prognosis

Treatment of small cell lung cancer (SCLC) is probably one of the great challenges of medical oncology owing to an increasing incidence in both men and women and a poor prognosis despite chemosensitivity (Ianuizzi and Scoggin, 1986; Hansen and Kristjansen, 1991). Serum markers have been proposed as a help in the management of SCLC during chemotherapy. In this setting, the most-established serum marker is the gamma–gamma isomer of a glycolytic enzyme referred to as neuron specific enolase (NSE) (Cooper et al; 1985; Jorgensen et al, 1989). High pretreatment levels of serum NSE are an indicator of extended tumour mass and poor prognosis (Cooper et al; 1985). Recently, two tumour markers detecting cytokeratins in the serum were proposed in the management of lung cancer (mainly non-SCLC): CYFRA 21-1 (Pujol et al, 1993, 1996*a*; Van Der Gaast et al, 1994), a cytokeratin fragment recognized by KS 19-1 and BM 19-21 antibodies, and TPS (Pujol et al, 1994), the specific M3 epitope of the tissue polypeptide antigen. It has been shown that a high pretherapeutic level of CYFRA 21-1 or TPS is an independent determinant of survival.

The prognostic value of the initial (pre-therapeutic) level of tumour markers is, therefore, well-established (Jorgensen et al, 1989; Pujol et al, 1993, 1994). However this level is not definitively fixed inasmuch as reversible states might be observed during therapy. Thus, the management of patients presenting SCLC might be helped by knowing the signification of each state (level) separately. These reversible variables are not only the serum level of markers (over or under the cut-off value) but also the response status of the disease as analysed using the World Health Organization (WHO) criteria (World Health Organization, 1979) (complete responders, partial responders and non-responders). For instance, it has been demonstrated that patients who benefit most from chemotherapy are those for whom a dramatic decrease in serum NSE level is observed (Jorgensen et al, 1989). However, the significance of this decrease in relation to patient outcome is unknown and thus is of poor clinical use as yet.

The homogeneous Markov model is a tool able to evaluate the transition intensities between states which are: a high level of marker (over cut-off level), a low level of marker and, finally,

*Received 20 January 1998*
*Revised 5 September 1998*
*Accepted 25 September 1998*

*Correspondence to:* JL Pujol death (Kay, 1986). Thus, this model allows the analysis of the relationship between the level of the marker and the outcome by using serial tumour marker assessments. It makes it possible to ascertain that a high level of marker is also an indicator of poor prognosis regardless of the time of transition from a low level to a high level. Thus, the Markov model assumption is that prognosis depends on past observations only through the last observed level of the marker (Kay, 1986). This assumption seems realistic in the case of SCLC. The present study deals with applying the homogeneous Markov model analysis to NSE, CYFRA 21-1, TPS serum levels and tumour response status on the one hand and their relationship with survival on the other. This study produced different transition intensities, particularly the instantaneous mortality hazard, of each marker, level by level.

### PATIENTS AND METHODS

### Patients

Fifty-two consecutive patients with pathologically confirmed SCLC as defined by the WHO classification (World Health Organization, 1982) were prospectively entered in the study. Performance status (PS) was estimated according to the Eastern Cooperative Oncology Group (ECOG) and the percentage of weight loss during the previous 4 months was recorded. Staging was carried out by exhaustive procedures according to the 4th edition of the *Union Internationale Contre le Cancer* (UICC) tumour node metastases (TNM) classification (Sobin et al, 1987), the American Thoracic Society map of regional pulmonary nodes (Tisi et al, 1982) and Mountain's stage grouping. In order to establish the disease stage, the following investigations were applied: clinical examination, standard chest roentgenography, computed tomographic (CT) scan of chest, upper abdomen and brain, fibreoptic bronchoscopy, and bone scanning. This staging procedure resulted in the classification of the disease into one of the two following groups: (1) limited disease, a disease confined to one hemithorax including mediastinal lymph nodes and/or supraclavicular lymph nodes; (2) extensive disease, defined as having the opposite criteria to limited disease. Patients with malignant pleural effusion were considered as having extensive disease. There were 27 (52%) patients suffering from limited disease and 25 from extensive disease.

The following pretreatment characteristics were also recorded: weight loss during the 3 months preceding the diagnosis, serum sodium (lower normal value 135 mmol $l^{-1}$), serum albumin (lower normal value 32 g $l^{-1}$), lactate dehydrogenase (LDH, upper normal value 600 U $l^{-1}$), alkaline phosphatase (upper normal value 125 U $l^{-1}$), blood leukocyte count (upper normal value 10 000 $\mu l^{-1}$) and performance status according the ECOG scale (63% had a PS 0 or 1 and 19 a PS 2). The complete panel of serum markers tested is described later.

### Sampling method

A blood sample was taken from each patient at presentation, and then at each other cycle visit (every 7 weeks). Following the treatment period, serum samplings were done during routine 3-monthly follow-up visits which consisted of a medical examination, chest X-ray and tumour marker assessment. The median number of marker level assessments was 4. However, due to the behaviour of SCLC (frequent relapses requiring readmission or new treatments, readmission for toxic events, etc.) some patients did not have samples taken as regularly as indicated above. The serum was separated and stored at – 190°C until tested. Serums were assayed blind of any clinical information; thus treatment decisions were taken without knowledge of serum marker levels.

### Biochemical measurements

CYFRA 21-1™ is a cytokeratin fragment 19 recognized by KS 19-1 and BM 19–21 monoclonal antibodies [mAb] (Centocor Diagnostics, Malvern, PA, USA and Cis Biointernational, Gif/Yvette, France). This solid phase immuno-radiometric assay based on the two-site sandwich method has been previously decribed in detail (Pujol et al, 1993). Briefly, in this method the cytokeratin 19 is recognized by two mouse MoAb, KS 19-1 and BM 19–21, directed against two different epitopes of a fragment of cytokeratin subunit 19, which is referred to as serum CYFRA 21-1. The calculated concentration of cytokeratin 19 is expressed in ng $ml^{-1}$.

TPS™ (BEKI Diagnostics AB, Bromma, Sweden) which detects the specific M3 epitope of the tissue polypeptide antigen (TPA) has been described extensively (Pujol et al, 1994). Briefly, TPS is a two-site immunoradiometric assay using two types of antibodies in excess: polyclonal anti-TPA horse antibodies bound to plastic beads and $^{125}I$ labelled mouse IgGl kappa anti-M3 MoAb. The latter antibody is raised against the M3 epitope of TPA which is specifically expressed by proliferating cells. The method of TPS titration has been described at length. The M3 concentration of the sample was determined using the results of construction of the standard curve and was expressed in U $l^{-1}$.

Serum NSE was measured by a radioimmunoassay (Cis Biointernational, Gif/Yvette, France). The respective upper limits of normal values were 3.6 ng $ml^{-1}$, 12.5 ng $ml^{-1}$ and 140 U $l^{-1}$ for serum CYFRA 21-1, serum NSE and serum TPS, respectively. These cut-off values have been predifined in previously published studies (Cooper et al, 1985; Pujol et al, 1993, 1994). They correspond to the 90–95% specificity as determined in patients with nonmalignant pulmonary disease.

### Treatment

For patients with extensive SCLC disease, chemotherapy consisted of a cyclophosphamide–4′-epidoxorubicin–etoposide and cisplatin combination given for a minimum of four cycles. Thoracic radiotherapy and prophylactic cranial irradiation were applied to patients with a complete response and to patients with partial response if residual tumour was confined to the chest at the end of the six-cycle chemotherapy programme. Treatment in patients with limited disease consisted of the same chemotherapy combination alternating with thoracic radiotherapy after cycle 2. Three courses of radiotherapy were given as a split course modality with concomitant prophylactic cranial irradiation during the third course. After the sixth cycle, chemotherapy was maintained only in those patients with residual disease on CT-scan whatever the pretherapeutic tumour status was.

### Response assessment

Indicator lesions were predefined at time of inclusion on CT-scan documents. Restaging was done at the end of the induction chemotherapy and every other cycle, thereafter WHO

 © Cancer Research Campaign 1999

recommendations on response reporting were applied (World Health Organization, 1979).

Our response assessment procedure has been described in detail (Pujol et al, 1996*b*). Briefly, tumour response for the chest, the liver, the adrenal glands and the brain were analysed by CT-scan assessing two-dimensional, measurable indicator lesions.

Calculation of response rate was done as follows:

$$\text{response (\%)} = \frac{(\text{inital D} \times \text{initial D}') - (\text{final D} \times \text{final D}')}{(\text{initial D} \times \text{initial D}')} \times 100,$$

where D and D′ are the longest perpendicular diameters of the indicator lesions. A complete response was defined as the complete disappearance of all lesions; a partial response was defined as equal to, or greater than, a 50% reduction in the product of the two longest perpendicular diameters of the indicator lesions for measurable tumours, or an improvement equal to or greater than 50% for evaluable-only tumours. Stable disease was defined as a less than 50% reduction and a less than 25% increase in this product. Finally, progressive disease was defined as equal to or greater than a 25% increase in this product, or appearance of new lesions. As published recently (Pujol et al, 1996*b*), the definition of a complete response included fibreoptic bronchoscopy data: a complete response was considered as a complete disappearance of all endo-bronchial lesion, or a non-specific scar, such as a minimal obstruction of a segmental bronchus with a negative bronchial re-biopsy.

Lesions identified by bone scanning were considered as evaluable-only. Tumour response was assessed according to the WHO criteria: disappearance of all lesions lasting for a minimum of 4 weeks resulted in a complete response. Appearance of new lesions was considered as progression. Other cases were considered as stable disease.

Finally, we defined the overall response as follows: where multiple indicator lesions were assessed, all individual results were taken into account. When discrepancies were observed in results obtained by different measurable indicator lesions, the worst results were considered overall according to the WHO recommendations (World Health Organization, 1979). On the other hand, a stable result obtained by assessing an evaluable lesion did not detract from a partial response in measurable lesions but reduced a complete response in measurable lesions to partial response overall. For instance, if the restaging demonstrated a partial response for a measurable indicator lesion on CT scan and a stable disease for bone metastasis evaluated by bone scan, the overall response was classified as a partial response. If in the total of responses per organ site there were equal or greater numbers of complete plus partial responses than of no change, the overall response was partial.

**Table 1** Cut-off level for each marker analysed

| | State 0 | State 1 |
|---|---|---|
| Serum NSE[a] | ≤ 12.5 ng ml$^{-1}$ | > 12.5 ng ml$^{-1}$ |
| | ≤ 25 ng ml$^{-1}$ | > 25 ng ml$^{-1}$ |
| Serum CYFRA 21-1 | ≤ 3.6 ng ml$^{-1}$ | > 3.6 ng ml$^{-1}$ |
| Serum TPS | ≤ 140 U l$^{-1}$ | > 140 U l$^{-1}$ |
| Response status[b] | CR or PR | NC |

CR, complete response; PR, partial response; NC, no change; PD, progressive disease. [a]In addition to the usual serum NSE threshold level of 12.5 ng ml$^{-1}$, a higher threshold, 25 ng ml$^{-1}$ was tested. [b]For the model using the response status, the absorbing state (state 2) was considered as either a progressive disease or death.

### Description of homogeneous Markov model

With conventional prognostic studies, patients are classified into different subgroups according to the value of a given variable recorded at time of origin of the study. Such a variable is referred to as a time-independent variable. This is the case with most of the pretreatment variables known to influence significantly the outcome of SCLC patients, e.g. metastatic status or performance status. Survival analysis consists of the calculation of the risk of death which affects a patient classified in a given subgroup. On the other hand, the Markov model allows the analysis of the prognostic influence by calculating a hazard ratio of a variable which can take different values for a given patient over a period of time. This type of variable is referred to as a time-dependent (or reversible) variable. The level of a tumour marker generally belongs to this category. In this model, for each evaluation the patients are classified into one of the following three states: dead (state 2, absorbing state as there is no transition from it), normal level of the marker (state 0) and high level of the marker (state 1). The last two states are called transient states as a given patient can move from the normal to the high level and conversely.

Transition intensities which link the transient states are conventionally denoted by $\lambda_{01}$ (transition from normal to high level state) and $\lambda_{10}$ (transition from a high to a low level state) whereas the risk of death is denoted by $\mu_0$ (risk of death from a normal level) and $\mu_1$ (risk of death from a high level). The stochastic structure of the Markov model is specified by its transition intensities according to the formula defined in the appendix (Kalbfleish and Prentice, 1980; Kay, 1986; Grüger et al, 1991).

Inasmuch as none of these transition intensities are time-dependent, the model is referred to as a time-homogeneous Markov model. The matrix of transition intensities is defined in the Appendix. The respective values of the states 0 and 1 for each analysed, reversible variable are described in Table 1. In addition to the usual serum NSE threshold level of 12.5 ng ml$^{-1}$, a higher threshold, 25 ng ml$^{-1}$, was tested in order to assess the validity of the model.

### Estimation method

We considered that the changes in marker levels were not concomitant with the sampling time. The hypothesis was that this change could have occurred at any time during the time interval between two serum tests. To ascertain the homogeneous assumption of the Markov model the transition intensities were analysed during the period following the induction therapy. Therefore, the chosen date of origin was the end of the second chemotherapy cycle. A detailed estimation method is reported in the Appendix.

### Test hypothesis

In order to test whether or not survival depends on the marker levels we used the likelihood ratio statistic $LR = 2\log L(\lambda_{01}, \lambda_{10}, \mu_0, \mu_1)/L(\lambda_{01}, \lambda_{10}, \mu_0, \mu_0)$, which under $H_0$: $\mu_0 = \mu_1$ has an approximatively $\chi^2$ distribution with one degree of freedom (Kay, 1986; Gentleman et al, 1994).

© Cancer Research Campaign 1999

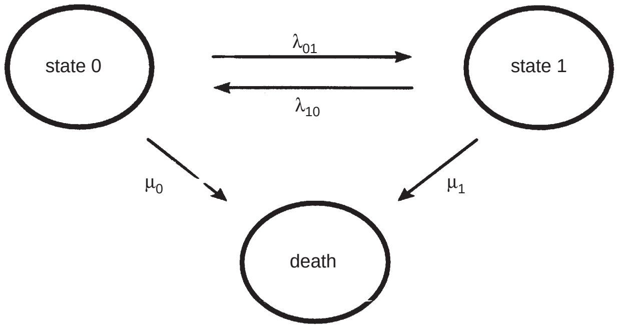

**Figure 1** State diagram of the homogeneous Markov model. States 0 and 1 are defined as the respective levels of the reversible variable (see Table 1). State 2 is the absorbing state (death). The possible transitions are shown by arrows. Transition between reversible states are characterized by the $\lambda$ transition intensity. Otherwise, instantaneous mortality hazards are characterized by $\mu$

**Table 2** Number of observed transitions

| Model | From state | To state | | | |
|---|---|---|---|---|---|
| | | 0 | 1 | 2 | Censored |
| NSE (12.5 ng ml$^{-1}$) | 0 | – | 18 | 17 | 8 |
| | 1 | 20 | – | 26 | 1 |
| CYFRA 21-1 | 0 | – | 5 | 32 | 8 |
| | 1 | 0 | – | 10 | 1 |
| TPS | 0 | – | 8 | 28 | 8 |
| | 1 | 3 | – | 15 | 1 |
| Tumour status | 0 | – | 0 | 30 | 7 |
| | 1 | 13 | – | 7 | 0 |

### Prognosis

The main purpose was to assess the respective effect of a high and a low marker level on prognosis at any sampling time $t$. This can be done via the transition matrix (Kay, 1986) defined by $P(s-t) = e^{Q(s-t)}$ and estimated by $\hat{P}(s-t) = e^{\hat{Q}(s-t)}$. This matrix gives the probabilities of being in one of the states described in Figure 1 at time $s$, taking into account the state occupied at time $t$. According to the homogeneity assumption, the $h$, $k$ entry $P_{hk}(d)$ is also the probability of being in state $k$, providing that the patient was in state $h$, $d$ weeks previously ($d$ might represent the time elapsed since the last sampling). Thus $P_{hk}(d)$ is an indicator of prognosis for the future outcome.

### Adjustment of Markov model using co-variables

In addition to the serum marker titrations we also recorded the status of the patient regarding other pre-treatment co-variables such as sex, performance status, nodal status, etc. Having obtained the Markov model describing the transition intensities linking the different states of a patient according to the level of the marker, the question is whether or not transition intensities are influenced by these putative co-variables. To answer this specific question the Markov model was adjusted using a stepwise regression (Kalbfleish and Lawless, 1985). In this procedure, co-variables were entered and removed according to the benefit or loss they brought to the likelihood as assessed by a likelihood ratio statistic. This process allowed all confounding variables to be removed from the model according to their correlation with the selected set of regression in the final model. By the end of this regression analysis, the transition intensity and the variables which statistically influence the transition have been described.

### RESULTS

### Three-state Markov model analysis using tumour markers and response status

In Table 2 the observed transitions are described. Table 3 shows the four Markov three-state models and the corresponding transition rate parameters (transition intensities and mortality hazards) of each reversible variable. Definition of state of disease and transition rates are shown in Figure 1.

The most striking model is the one using the 12.5 ng ml$^{-1}$ threshold for serum NSE. The mean time to move out of state 1 (high NSE level) was $1/(\lambda_{10} + \mu_1)$ = 123 days. At that time, the probability that the opposite reversible state (alive with low NSE level) was reached, was $\lambda_{10}/(\lambda_{10} + \mu_1)$ = 44%, demonstrating the reversible property of state 1; on the other hand, the probability that the patient died was $\mu_1/(\lambda_{10} + \mu_1)$ = 56%. The mean time to move out of state 0 (low NSE level) was 238 days. The relative risk of death for state 1 (namely mortality hazard $\mu_1$) was about 2.24 times greater in comparison with that for state 0 (mortality hazard $\mu_0$). In this setting, the likelihood ratio statistic demonstrated that this difference was significant. Thus, a patient in state 1 (high NSE level) was at high risk of death. This suggests that the observation of an increase of NSE at any time during treatment (according to the homogeneous assumption) was strongly associated with a worse prognosis. The respective transition parameters obtained with the 12.5 and 25 ng ml$^{-1}$ thresholds suggested a gradual increase in risk inasmuch as the $\mu_1/\mu_0$ ratio was 2.24 for the lower threshold and 4.67 for the higher.

**Table 3** Transition intensities and mortality hazard (standard errors) among states for each marker studied[a]

| Parameters | NSE 12.5 ng ml$^{-1}$ | NSE 25 ng ml$^{-1}$ | CYFRA21-1 | TPS | Tumour response |
|---|---|---|---|---|---|
| $\lambda_{01}$ | 2.166 (0.512) | 0.56 (0.21) | 0.399 (0.178) | 0.687 (0.243) | [b] |
| $\lambda_{10}$ | 3.521 (0.793) | 2.08 (1.21) | [c] | 1.277 (0.740) | 11.631 (0.335) |
| $\mu_0$ | 2.046 (0.498) | 2.23 (0.42) | 2.552 (0.452) | 2.405 (0.455) | 3.054 (0.056) |
| $\mu_1$ | 4.577 (0.907) | 10.42 (2.77) | 7.872 (2.548) | 6.385 (1.680) | 6.263 (0.242) |
| LR statistic | 6.688 ($P$ < 0.01) | 18.11 ($P$ < 0.001) | 7.578 ($P$ < 0.01) | 7.982 ($P$ < 0.005) | 2.1 ($P$ = 0.12) |

LR, likelihood ratio. [a]The table entries are the transition intensities and mortality multiplied by $10^3$. [b]We did not estimate $\lambda_{01}$ transition intensity for tumour response owing to the lack of observed transition from state 0 to state 1. [c]We did not estimate $\lambda_{10}$ transition intensity for CYFRA 21-1 owing to the lack of observed transition from state 1 to state 0.

 © Cancer Research Campaign 1999

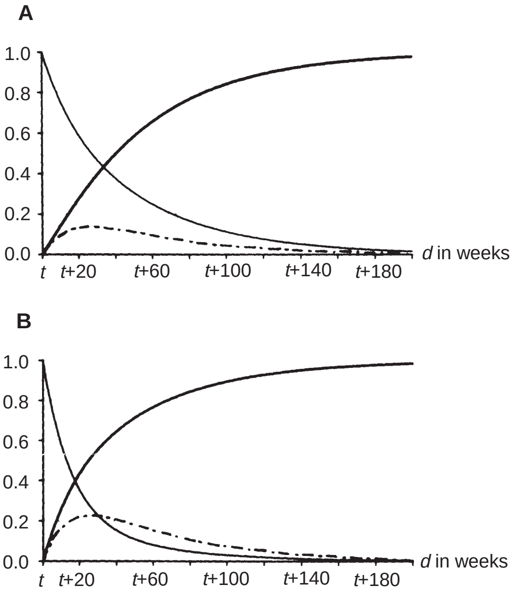

**Figure 2** Effect of serum NSE level on the probability that can be prognosed *d* weeks after sampling at time *t* (taking into account the 12.5 ng $ml^{-1}$ threshold). Each curve represents the probability at time *t* + *d* weeks: thick line, probability of death; thin line, probability of remaining in the same reversible state; dotted line, probability of changing to the opposite level. **(A)** for a patient in state 0 at time *t* **(B)** for a patient in state 1 at time *t*

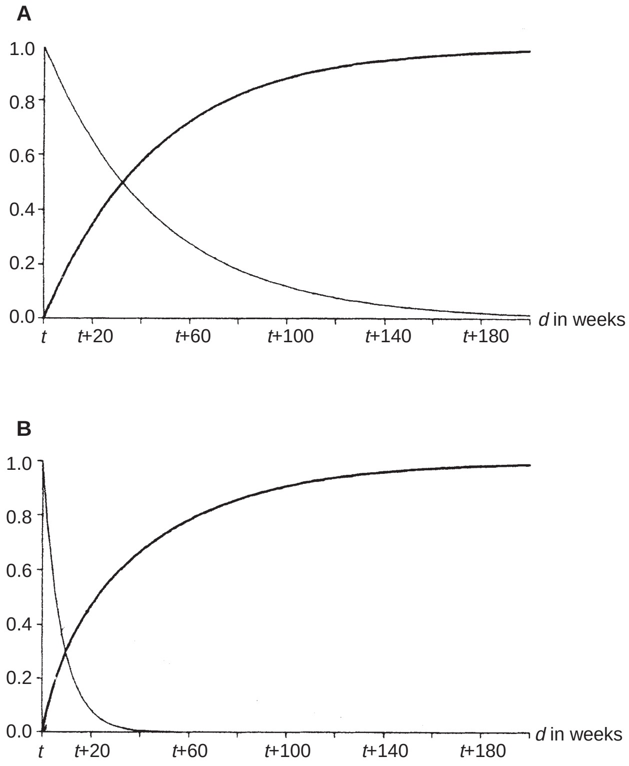

**Figure 3** Effect of tumour response status level on the probability that can be prognosed *d* weeks after sampling at time *t*. Each curve represents the probability at time *t* + *d* weeks: thick line, probability of death; thin line, probability of remaining in the same reversible state; dotted line, probability of changing to the opposite level. **(A)** for a patient in state 0 at time *t*, **(B)** for a patient in state 1 at time *t*

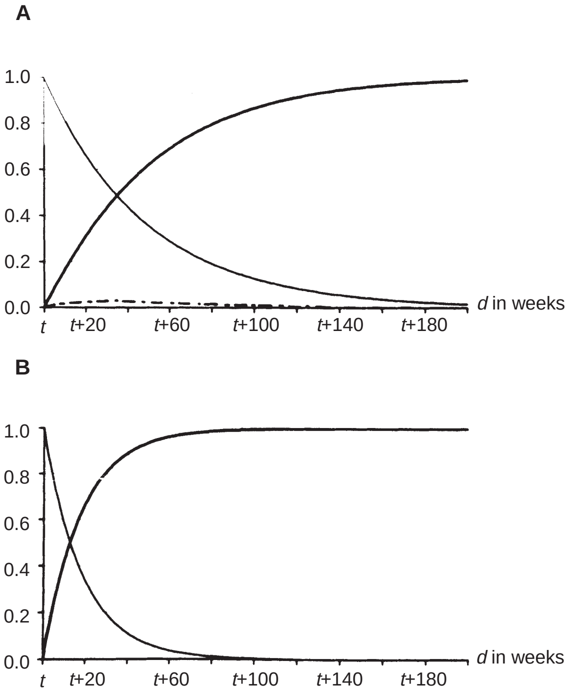

**Figure 4** Effect of serum CYFRA 21-1 level on the probability that can be prognosed *d* weeks after sampling at time *t*. Each curve represents the probability at time *t* + *d* weeks: thick line probability of death; thin line, probability of remaining in the same reversible state; dotted line, probability of changing to the opposite level. **(A)** for a patient in state 0 at time *t*, **(B)** for a patient in state 1 at time *t*

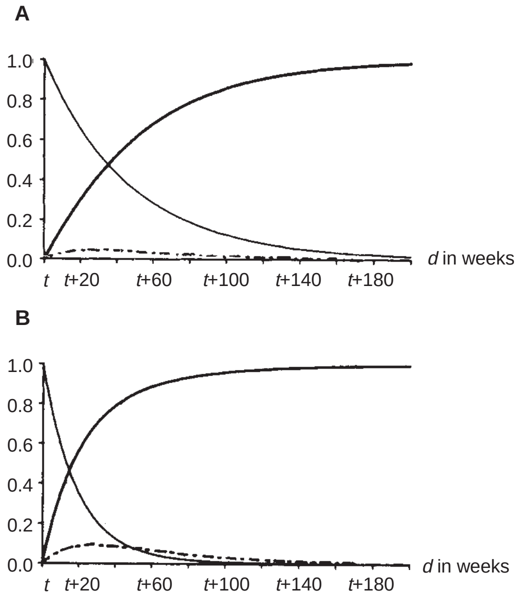

**Figure 5** Effect of serum TPS level on the probability that can be prognosed *d* weeks after sampling at time *t*. Each curve represents the probability at time *t* + *d* weeks: thick line, probability of death; thin line, probability of remaining in the same reversible state; dotted line, probability of changing to the opposite level. **(A)** for a patient in state 0 at time *t*, **(B)** for a patient in state 1 at time *t*

© Cancer Research Campaign 1999

**Table 4** Hazard ratio for co-variables influencing the transition intensities of the Markov model constructed using the NSE level (threshold 12.5 ng ml$^{-1}$)

| Variables | Transitions | Hazard ratio | Asymptotic 95% CI | *P*-value |
|---|---|---|---|---|
| Sex | $\lambda_{01}$ | 5.13 | [– 1.27; 11.53] | 0.021 |
| LDH | $\lambda_{01}$ | 0.16 | [– 0.16; 0.48] | 0.021 |
| T status | $\lambda_{01}$ | 0.31 | [– 0.02; 0.63] | 0.033 |
| Age | $\mu_{02}$ | 3.24 | [0.01; 6.47] | 0.017 |
| N | $\lambda_{10}$ | 3.61 | [0.26; 6.96] | 0.005 |
| N | $\mu_{12}$ | 2.53 | [0.41; 4.64] | 0.029 |

Analysing CYFRA 21-1, the three-state Markov model again demonstrated a significant difference in mortality hazards as patients in state 1 (alive with a high CYFRA 21-1 level) had an instantaneous risk of death treble that of patients in state 0. Again the likelihood statistic was significant. However, using the well-established 3.6 ng ml$^{-1}$ CYFRA 21-1 threshold, the state of the marker did not demonstrate a reversible property: the mean time to move out of state 1 was 128 days. This movement only depended on the probability of death. In other words, the probability was zero of a patient returning to state 0 having once entered state 1. The mean time to move out of state 0 was 344 days. Although non-nil, the probability of the transition reaching state 1 was only 14% and thus the probability that transition was due to death of the patient was 86%.

Transition parameters of TPS also demonstrated a higher mortality hazard for patients in state 1 (alive with high TPS level) in comparison with those patients in state 0 (likelihood ratio test $P < 0.005$). Similar to CYFRA 21-1, passages from state 0 to 1, and conversely, were of low intensity. Thus, the probability of a patient returning to state 0, having once reached state 1, was low. This weakens the use of TPS as a reversible tumour marker in SCLC.

Finally, the three-state Markov model analysing tumour response status considered responder patients as occupying state 0, whereas non-responders occupied state 1 and patients with either a progressive disease and patients who died occupied state 2 (absorbing state). Mortality hazards differed with patients in state 1 having the higher risk. The relative risk of death for state 1 (namely mortality hazard $\mu_1$) was about 2.1. However, in this setting, the likelihood ratio statistic did not demonstrate statistical significance. This was probably due to the low number of transitions (Table 2). There was no true reversible property of states 0 and 1 as the probability $\lambda_{10}$ was 0.

### Prognostication of outcome using the data obtained from Markov models

As a complement to the instantaneous transition rates, inferent statistics produced the probability functions charted in Figures 2 to 5 taking into account the state of the marker at time *t*. Each curve represents the probability function which prognosticates *d* weeks after sampling the occupied state (opposite transient state, death or stationary position in the same state). As shown in Figure 2 regarding the three-state Markov model analysing the NSE level, these probabilities differed according to the state occupied at time *t*. Where the patient was in state 0 (alive with low NSE level) at time *t*, the probability of being stationary at this state decreased slowly with time, whereas the probability of death progressively increased. The lowest probability was the transition to state 1. Graphically, this last transition appeared to occur mainly during the first weeks following the sampling whereas, after this time, patients were almost exclusively exposed to the risk of death as the only possible transition. Conversely, when one considers a patient in state 1 at time *t*, the probability of death (1 — probability of survival) dramatically increased, a transition which explains the rapid decrease of the probability of remaining stationary at state 1. However, a non-nil probability of changing from state 1 to the opposite transient level state 0 was observed suggesting that, however infrequently, patients in state 1 might still return to state 0. Similar conclusions can be drawn regarding the three-state model constructed using the tumour response status (Figure 3).

Less informative are the probability functions constructed using the three-state Markov model analysing CYFRA 21-1 (Figure 4). Whatever the considered state occupied at time *t* the probability of death was roughly equal to 1 — the probability of remaining stationary at the state occupied at time *t* owing to the small number of transitions observed between transient states. The mean survival was therefore reached when the probability function of being in the same state decreased to 50%. Again, this demonstrated the lack of true reversible status of CYFRA 21-1 in SCLC. Almost similar conclusions can be drawn regarding the three-state model constructed using TPS levels (Figure 5).

### Adjustment of the Markov model using co-variables

As the aim of this study was to investigate the prognostic value of markers, only the Markov model using the serum NSE titrations was adjusted as NSE was the only model of interest in this setting (using the 12.5 ng ml$^{-1}$ threshold). Stepwise regression analysis demonstrated that the $\mu_1$ transition intensity was significantly influenced by the nodal status co-variable inasmuch as patients with a positive mediastinal nodal status have a death hazard ratio from a state ‘high serum NSE level’ of 3.6. On the other hand, patients over 63 years proved to have a death hazard ratio of 2.5 from a state of ‘normal serum NSE level’ ($\mu_0$; Table 4).

### DISCUSSION

Among the different clinical applications of tumour markers in human malignancies, prognostication of the outcome might be regarded as an important issue (Bates, 1991). For lung cancer, decisions regarding therapy are influenced not only by pretreatment prognostic determinants (tumour status, stage of the disease, histology) but also by variables occurring during the course of the disease, for instance, the appearance of new lesions or occurrence of life-threatening toxic events (Kay, 1986; Andersen et al, 1991). The pretreatment value of NSE has been demonstrated as being a prognostic determinant in SCLC (Cooper et al, 1985). Patients with a high serum NSE level at time of diagnosis prove to have a shorter survival than patients with a serum NSE equal to or less than the cut-off value. These results, together with data produced by other studies for larger populations, suggest that a patient with lung cancer associated with a high serum NSE level is at higher risk of death. The question remains as to the clinical use of such an observation. From the research point of view, the precise characterization of prognostic factors is important when analysing survival data produced, for instance, by two different treatments. From a patient-by-patient point of view, however, awareness of baseline prognostic variables, except stage of disease, might be considered as a secondary end-point because it does not influence treatment decision, namely chemotherapy. In addition, variables such as tumour

 *© Cancer Research Campaign 1999*

marker levels or tumour status are not definitively fixed at time of presentation and changes in the level might occur as a result of both the course of the disease and the effect of therapy. This is not predicted by the classical Cox's model (Cox, 1972).

Different methods have been proposed in order to define the specific clinical effect of the change in a marker level during therapy of SCLC. One of them consisted of analysing concordance between modifications of marker level and modifications of tumour status. Several studies have observed similar behaviour of tumour mass and NSE level during chemotherapy of SCLC (Cooper et al, 1985; Jorgensen et al, 1988). It has been demonstrated that the serum NSE level decreases under cut-off value at the time when tumour response is demonstrated by tumour measurement of indicator lesions. Three comments might be raised in analysing such data: first, if the biological decrease is concordant with the tumour response assessment, the former parameter might be futile as the second is required according to the World Health Organization Recommendations in Reporting Results of Cancer Treatment. Secondly, some patients have a persistent high serum NSE level although they benefited from a tumour response; whether or not this condition is different from the general one (concordant decrease of NSE level and tumour mass) is unknown. Third, the concomitant analysis of tumour marker level and tumour mass does not answer the main question regarding the relationship between kinetics of reversible disease indicators and survival.

The Cox's model method has been extended to the analysis of time-dependent covariates, i.e. reversible variables (Andersen et al, 1991). Unlike the classical Cox's model which analyses variables definitively fixed at the time of origin (Cox, 1972), this method considers that a patient could move from one stratum to another, according to the value of the time-dependent co-variable. It allows the calculation of the death risk linked to the strata but does not predict the changes of the time-dependent variable as a function of time. In other words, it gives prognostic information stratum-by-stratum but no information about the probabilities of passage from one state to the other. In addition, tumour markers or tumour disease status are analysed only at baseline time, and then at rather infrequent follow-up times, due to the nature of the disease. Thus, the analysis of such an incomplete observation in Cox's model might be hazardous. On the other hand, the three-state Markov model, as described in this paper, allows an estimation of probability for a patient to be in various states, even in the case where there is incomplete information regarding the reversible disease indicators (Gentleman et al, 1994).

In our study we analysed the three-state Markov model of tumour response assessment and three different tumour markers. Patients have been studied longitudinally. They all received a cisplatin-based chemotherapy. The application here of the Markov model using marker serum levels in SCLC followed the methodology proposed by Kay (1986) in the setting of Markov analysis of α-foeto-protein in hepatocellular carcinoma. In SCLC, the homogeneous Markov assumption is supported by the short survival with high rate of transitions from state to state occurring early after diagnosis, even during chemotherapy. Thus, one may assume that the Markov model is homogeneous. The validity of the model is also suggested by the increase in risk $\mu_1/\mu_0$ observed when a higher serum NSE cut-off level is chosen. Using the Markov model to analyse CYFRA 21-1 or TPS allows two conclusions: first, the mortality hazard for a patient who has an elevated serum cytokeratin level is significantly higher when compared with one of the patients with a low cytokeratin level. Second, the probabilities of transition from one state to the other (high or low serum cytokeratin level) are low, particularly in the case of CYFRA 21-1. Thus, it does not seem useful to make serial measurements of these markers during treatment and follow-up of SCLC as the essential prognostic information lies in the baseline titration with almost no change in state thereafter.

Conversely, the three-state Markov models of serum NSE and tumour response assessment are informative about the possible use of these reversible variables. If one considers a patient with a high NSE level at any time during the treatment course three indications can be found: the patient is at higher risk of death in comparison with the opposite transient level (serum NSE less than cut-off value); the mean time to move out of this state is short; the risk of death represents the main explanation for this instability but the patient still has a good probability (44%) of returning to the lower risk state. Almost similar observations are made for a patient disclosing a non-responder disease status. According to the regression analysis, the hazard ratio of risk of death according to a high or low level of NSE is influenced by few co-variables, namely nodal status and age. This suggests the relative independence of the prognostic value of NSE in SCLC and it might justify salvage therapy in patients who experience either an increase in NSE level or progression of the disease. The homogeneous assumption of the Markov model supposed that a patient who enters for the first time an elevated state at time *t* has the same prognosis as the one who has more than one transition in his previous history before entering this state. From a clinical point of view, this assumption might be realistic in the case of SCLC. The validity of the Markov model is based on the number of transitions (which is comparable to the number of events) rather than the number of patients. This number is 81 for the NSE Markov model. However, larger multi-centre populations of SCLC will be needed in order to confirm the results of our study.

One important remaining question belongs to the relevance of the observation made using the Markov model in clinical practice. There is, currently, a hope that new drugs such as topoisomerase I inhibitors and taxanes might be active treatments for patients who relapse from SCLC and were initially sensitive to more conventional chemotherapy. Therefore, tools able to monitor correctly the disease after induction therapy might be required in the near future.

The three-state Markov models analysing serum NSE or tumour response assessment in SCLC make it possible to define the time to transition, transition intensities and mortality hazards according to the different states of these reversible disease indicators. Markov analysis takes into account the incomplete nature of the observation of these variables which are not monitored continuously. It therefore confers clinical usefulness on both serum NSE follow-up and tumour reassessment by measuring their relationship with prognosis.

### ACKNOWLEDGEMENTS

This paper was supported by a grant from the French League Against Cancer (Herault and Aude committees) and the 'Groupement des Entreprises Françaises dans La Lutte contre le Cancer'. The authors wish to thank Mrs Jo Baïssus for help in preparing the manuscript and Dr L Saffont for help in data management.

### REFERENCES

Andersen PK, Hansen LS and Keiding N (1991) Assessing the influence of reversible disease indicators on survival. *Stat Med* **10**: 1061–1067

Bates SE (1991) Clinical applications of serum tumour markers. *Ann Intern Med* **115**: 623–638
Cooper EH, Splinter TAW and Brown DA (1985) Evaluation of a radio-immunoassay for neuron-specific enolase in small cell lung cancer. *Br J Cancer* **52**: 333–338.
Cox DR (1972) Regression models and life tables. *J Roy Stat Soc B* **34**: 187–220
Gentleman RC, Lawless JF, Lindsey JC and Yan P (1994) Multi-state Markov models for analysing incomplete disease history data with illustrations for HIV disease. *Stat Med* **13**: 805–821
Grüger J, Kay R and Schumacher M (1991) The validity of inferences based on incomplete observations in disease state models. *Biometrics* **47**: 595–605
Hansen HH and Kristjansen PEG (1991) Chemotherapy of small cell lung cancer. *Eur J Cancer* **27**: 342–349
Iannuzzi MC and Scoggin CH (1986) State of the art: small cell lung cancer. *Am Rev Res Dis* **134**: 593–608
Jørgensen LGM, Osterlind K, Hansen HH and Cooper EH (1988) The prognostic influence of serum neuron specific enolase in small cell lung cancer. *Br J Cancer* **58**: 805–807
Jørgensen LGM, Hansen HH and Cooper EH (1989) Neuron specific enolase, carcinoembryonic antigen and lactate dehydrogenase as indicators of disease activity in small cell lung cancer. *Eur J Cancer Clin Oncol* **25**: 123–128
Kalbfleish JD and Prentice RL (1980) *The Statistical Analysis of Failure Time Data*. Wiley: New York
Kalbfleish JD and Lawless JF (1985) The analysis of panel data under a Markov assumption. *JASA* **80**: 863–871
Kay R (1986) A Markov model for analysing cancer markers and disease states in survival studies. *Biometrics* **42**: 855–865
Pujol JL, Grenier J, Daurès JP, Daver A, Pujol H and Michel FB (1993) Serum fragment of cytokeratin subunit 19 measured by CYFRA 21-1 immuno-radiometric assay as a marker of lung cancer. *Cancer Res* **53**: 61–66
Pujol JL, Cooper EH, Grenier J, Purves DA, Lehmann M, Ray P, Dan Aouta M, Bashir M, Godard P and Michel FB (1994) Clinical evaluation of serum TPS in non-small cell lung cancer. *Eur J Cancer* **30A**: 1768–1774
Pujol JL, Grenier J, Parrat E, Lehmann M, Lafontaine M, Quantin X and Michel FB (1996*a*) Cytokeratins as serum markers in lung cancer: a comparison of CYFRA 21-1 and TPS. *Am J Res Crit Care Med* **154**: 725–732
Pujol JL, Parrat E, Lehmann M, Gautier V, Daurès JP, Michel FB and Godard P (1996*b*) Lung cancer chemotherapy: methods of response evaluation and response–survival relationship. *Am J Res Crit Care Med* **153**: 243–249
Sobin LH, Hermanek, P and Hutter, RVP (1987) *TNM Classification of Malignant Tumours*, 4th edn. UICC: Geneva
Tisi GM, Friedman PJ, Peters RM, Pearson G, Carr D, Lee RE and Selawry O (1982) American Thoracic Society: clinical staging of primary lung cancer. *Am Rev Res Dis* **125**: 659–664
Van Der Gaast A, Schoenmakers CHH, Kok TC, Blijenberg BG, Cornillie F and Splinter TAW (1994) Evaluation of a new tumour marker in patients with non-small cell lung cancer: CYFRA 21-1. *Br J Cancer* **69**: 525–528
World Health Organization (1979) *WHO Handbook for Reporting the Results of Cancer Treatment*. WHO Offset Publication, 48: Geneva
World Health Organization (1982) The World Health Organization histological typing of the lung tumours, 2nd edn. *Am J Clin Pathol* **77**: 123–136

### APPENDIX

### Transition intensities which define the stochastic structure of the Markov model

$$\lambda_{hl}(t) = \lim_{\Delta \to 0} \frac{P\text{–[transition } h \to l \text{ in } [t, t+\Delta] \mid \text{state } h \text{ at } t],}{\Delta} \text{for } h, \text{l} = 0, 1 \text{ or } 1, 0,$$

$$\mu_h(t) = \lim_{\Delta \to 0} \frac{P[\text{transition } h \to \text{death in } [t, t+\Delta] \mid \text{state } h \text{ at } t],}{\Delta} \quad \text{for } h, \text{l} = 1, 2.$$

### Matrix of transition intensities

$$Q = \begin{pmatrix} -(\lambda_{01} + \mu_0) & \lambda_{01} & \mu_0 \\ \lambda_{10} & -(\lambda_{10} + \mu_1) & \mu_1 \\ 0 & 0 & 0 \end{pmatrix}$$

### Estimation method of the Markov model

If M referred to the number of serum assessments, $T_0, T_1, \ldots, T_M$ referred to the sampling dates and $Y_0, Y_1, \ldots, Y_M$ referred to the respective values of the marker transformed into a binary variable as described in Table 1. Let $T$ express the minimum time between survival and censoring date and $D$ express the censoring indicator ($D = 0$ for an individual right-censored, $D = 1$ for an observed death). Because serum was assayed blind of any clinical information, the known or unknown current marker state did not affect the probability of a patient being tested providing information not yet recorded. We also assumed that all right-censoring times were non-informative of the disease progression, i.e. did not depend on future behaviour.

According to the above assumptions, the likelihood of $\{M, T_0, Y_0, T_1, Y_1, \ldots, T_M, Y_M, D\}$ is proportional to the likelihood obtained if the number of serum tests and their times were preplanned. Thus our sampling plan is called non-informative for the disease process, according to Kalbfleish and Prentice (1980) and Grüger et al (1991).

Let $L(y_{i,j}; y_{i,j-1}, t_{i,j} - t_{i,j-1}) = L(y_{i,j}; t_{i,j}, y_{i,j-1}, t_{i,j-1} \ldots, t_{i,0}, y_{i,0})$ express the probability of making one transition from $Y_{i,j-1}$ to $Y_{ij}$ in time interval $[t_{ij-1}, t_{i,j}]$ given the past. Therefore, each individual record $\{m_i, t_i, 0, Y_{i,0}, t_{i,1}, Y_{i,1}, \ldots, t_{i,mi}, Y_{mi}, t_i, d_i\}$ contributes to the likelihood of the transition intensities ($l$, $m$) and is expressed as the following factor:

$$L_i = \prod_{j=1}^{j=m_i} L(y_{i,j}; y_{i,j-1}, t_{i,j} - t_{i,j-1})$$

$$(L(x_i; y_{i,m_i}, t_{i,m_i} - t_{i,m_i-1}))^{d_i} (1 - L(c_i; y_{i,m_i}, t_{i,m_i} - t_{i,m_i-1}))^{1-d_i}.$$

The likelihood function for all patients was $L(\lambda,\mu) = \Pi_i L_i$. The model parameters ($l$, $m$) were obtained by using the Newton–Raphson algorithm implemented on BMDP software. The prechosen starting values of this iterative procedure were the maximum likelihood estimators when one assumed that the first recorded time in a new state was the exact time of entry into that state, i.e. the quotient between $m_{hl}$, the total number of transitions from state $h$ to state $l$ and $T_h$, the total time spent in state $h$ by all individuals,

$$\lambda_{hl}^{(0)} = \frac{m_{hl}}{T_h}, \; \mu_h^{(0)} = \frac{m_{h,3}}{T_h}, \text{ for } h = 0 \text{ or } 1.$$

Given that a homogeneous three-state Markov process is currently in state $h$, the time until it moves out of that state has an exponential distribution with rate $(\lambda_{hl} + \mu_h)$, $l \neq h$, therefore the mean time for moving out of a current state $h$ is $1 / (\lambda_{hl} + \mu_h)$, $l \neq h$ and the probability that the change is to state $l$ equals $\lambda_{hl} / (\lambda_{hl} + \mu_h)$, $l \neq h$.

 *© Cancer Research Campaign 1999*

### Example of clinical data

| Date | NSE (12.5 ng ml$^{-1}$) | CYFRA 21-1 | TPS | Tumour status |
|---|---|---|---|---|
| 12-12-90 | State 1 | State 1 | State 1 | State 1 |
| 01-07-91 | State 1 | State 0 | State 1 | State 0 |
| 01-28-91 | State 0 | State 0 | State 1 | State 0 |
| 02-21-91 | State 0 | State 0 | State 1 | State 0 |
| 04-09-91 | State 0 | State 0 | State 1 | State 0 |
| 07-08-91 | State 0 | State 0 | State 1 | State 2 |
| 11-05-91 | State 1 | State 0 | State 1 | State 2 |
| 01-03-92 | State 2 | State 2 | State 2 | State 2 |

A 63-year-old women suffering from extensive-stage SCLC. Despite a chemotherapy-induced complete response lasting 5 months, relapses occurred and were responsible for death 54 weeks after the diagnosis. For the serum level NSE, the transition from state 0 to state 1 was an important prognostic determinant.

*© Cancer Research Campaign 1999*
